# Extracellular ATP induces apoptosis through P2X7R activation in acute myeloid leukemia cells but not in normal hematopoietic stem cells

**DOI:** 10.18632/oncotarget.13927

**Published:** 2016-12-13

**Authors:** Valentina Salvestrini, Stefania Orecchioni, Giovanna Talarico, Francesca Reggiani, Cristina Mazzetti, Francesco Bertolini, Elisa Orioli, Elena Adinolfi, Francesco Di Virgilio, Annalisa Pezzi, Michele Cavo, Roberto M Lemoli, Antonio Curti

**Affiliations:** ^1^ Department of Experimental, Diagnostic and Specialty Medicine, University of Bologna, Bologna, Italy; ^2^ European Institute of Oncology, Milan, Italy; ^3^ Department Biomedical and Neuromotor Sciences, University of Bologna, Bologna, Italy; ^4^ Department of Morphology, Surgery and Experimental Medicine, University of Ferrara, Ferrara, Italy; ^5^ Clinic of Hematology, Department of Internal Medicine (DiMI), University of Genoa, Genoa, Italy

**Keywords:** acute myeloid leukemia (AML), leukemic stem cell (LSC), P2×7R, ATP, apoptosis

## Abstract

Recent studies have shown that high ATP levels exhibit direct cytotoxic effects on several cancer cells types. Among the receptors engaged by ATP, P2×7R is the most consistently expressed by tumors. P2×7R is an ATP-gated ion channel that could drive the opening of a non-selective pore, triggering cell-death signal. We previously demonstrated that acute myeloid leukemia (AML) cells express high level of P2×7R. Here, we show that P2×7R activation with high dose ATP induces AML blast cells apoptosis. Moreover, P2×7R is also expressed on leukemic stem/progenitor cells (LSCs) which are sensitive to ATP-mediated cytotoxicity. Conversely, this cytotoxic effect was not observed on normal hematopoietic stem/progenitor cells (HSCs). Notably, the antileukemic activity of ATP was also observed in presence of bone marrow stromal cells and its addition to the culture medium enhanced cytosine arabinoside cytotoxicity despite stroma-induced chemoresistance. Xenotransplant experiments confirmed ATP antineoplastic activity *in vivo*.

Overall, our results demonstrate that P2×7R stimulation by ATP induced a therapeutic response in AML at the LSC level while the normal stem cell compartment was not affected. These results provide evidence that ATP would be promising for developing innovative therapy for AML.

## INTRODUCTION

Acute myeloid leukemia (AML) is a hematopoietic stem cell disorder characterized by clonal proliferation of myeloid precursors with an inhibition in differentiation, leading to accumulation of immature cells at various stages. Approximately 50% of leukemia patients survive their disease. The percentage of long-term survivors drops down to 5–15% in elderly patients. Disease relapse and toxicity of therapy, especially in elderly patients, represent today the major limiting factors in leukemia treatment. Despite the introduction of novel target therapies in the treatment of cancer, chemotherapy remains as primary option for AML patients. In this scenario, novel therapeutic approaches that aim to reduce toxicity and to improve the efficacy of treatment are expected to greatly improve long-term outcomes in leukemia patients.

ATP is the key energy currency as well as an ubiquitous extracellular messenger. Depending on its dose and the purinergic P2 receptor (P2R) subtype engaged, ATP can trigger many different cell responses, including cell proliferation, differentiation and apoptosis [1].

Over the past decade, various studies described a direct cytotoxicity of extracellular ATP on different tumor cell types, such as melanoma, glioma and colon cancer cells [1–3]. ATP acts binding fifteen different purinergic receptors but only five have been described to be involved in ATP direct tumor killing: P2×5R, P2×7R, P2Y1R, P2Y2R and P2Y11R. Among these, P2×7R is the most consistently expressed by tumor cells [4, 5].

P2×7R is an ATP plasma membrane ion channel that, upon transiently stimulation, behaves like a cation-selective channel permeable to Na^+^, K^+^ and Ca^2+^. It shows a growth-promoting activity, which seems to be indispensable for different cell types, such as T lymphocyte or primary mouse microglia cells [6–9]. One of the most interesting feature of the P2×7R trophic effect is its ability to promote survival and growth in absence of serum [10, 11]. Therefore, it is not surprising that malignant tumors overexpress this receptor. Several groups have described increased expression of P2×7R in prostate [12], breast and skin cancers [13, 14], neuroblastoma [15, 16], leukemia [17, 18] and thyroid papillary carcinoma [19]. This observation, supported by recent studies [20], suggested that P2×7R could have an important role in carcinogenesis and tumor progression [21]. However, P2×7R is an unusual receptor and its massive, unlike tonic low-level, stimulation by ATP leads to the formation of a non selective and permanently opened pore in the cell membrane, triggering cell death signals. Over the past years, several reports showed the possibility to induce tumor inhibition or killing by P2×7R activation [22, 23].

We previously reported that unlike normal CD34^+^ cells [24], activation of P2R signaling by ATP inhibits leukemic cell functions [18]. Since it has opposite effect on hematopoietic stem cells (HSCs) and leukemic cells, ATP is a good candidate for developing therapies with low toxicity on HSC compartment, which is essential for a complete hematologic recovery after therapy. In the present study, we extended our previous biological findings by testing the therapeutic potential of high dose of ATP *in vitro* and *in vivo*. We demonstrated that leukemic stem cells (LSCs) are sensitive to ATP treatment being induced to apoptosis while the same treatment did not affect normal HSC compartment. Moreover, the addition of ATP enhanced the antileukemic activity of cytosine arabinoside and counteracted stroma-mediated chemoresistance. In addition, we demonstrated the *in vivo* antileukemic effect of ATP treatment. Our data, combined with published studies, suggest the antitumor potential of purinergic-based drugs and propose P2×7R as target for development of therapeutic strategies in leukemia treatment.

## RESULTS

### P2×7R activation by ATP induces apoptosis of primary AML cells

**Figure 1:**
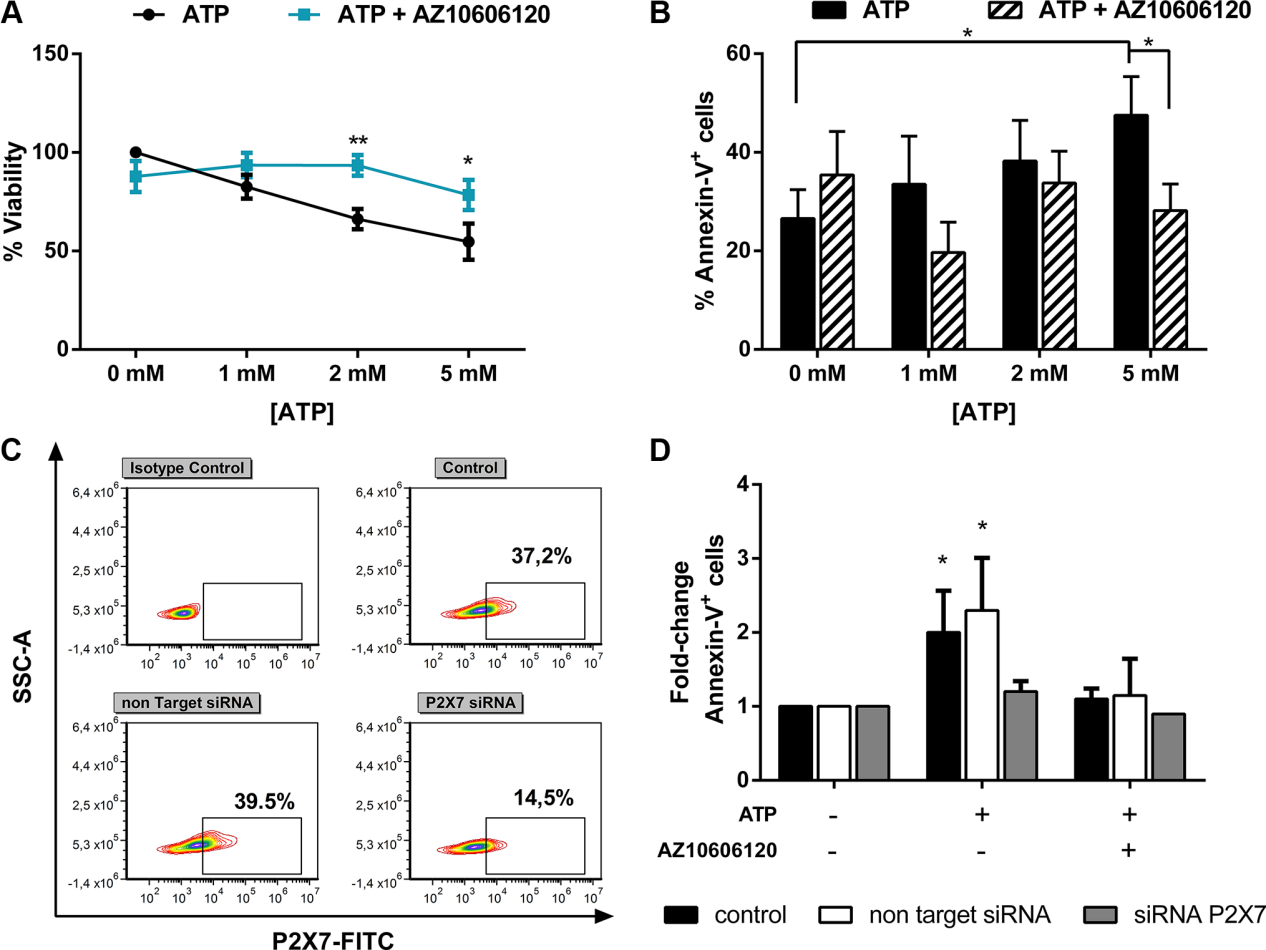
ATP triggers apoptosis of leukemia cells from AML patients via P2×7 activation Leukemic cells isolated from AML patients were treated for 48 h with increasing doses of ATP, with or without (w/o) 10 μM AZ 10606120. Data are represented as mean +/− SEM (**A**) CellTiter 96 Aqueous One Solution assay was used to detect viability (*n* = 14) and (**B**) Annexin V/PI staining was used to detect apoptosis (*n* = 23). (**C**–**D**) To inhibit P2×7 expression, AML cells were nucleofected with a Non Targeting control siRNA or with P2×7-specific siRNA. After overnight, cells were treated with 5 mM ATP for 24 h, with or w/o 10 μM AZ 10606120 (*n* = 4). Results are expressed as fold-change of Annexin-V^+^ cells respect to untreated cells, for each group (% Annexin-V^+^ cells: 22.4 ± 7% control, 19 ± 6% Non Targeting Control siRNA, 23.4 ± 9.6% P2×7 siRNA). (C) Representative flow cytometric analysis of P2×7 expression after siRNA treatment. *p < 0.05.

We first investigated whether ATP, via P2×7R activation, induces apoptosis in primary AML cells. In line with previous report [23], we showed that ATP exerted direct cytotoxicity on AML cells reducing cell viability in a dose dependent manner. This effect is inhibited by P2×7R blockage through the addition of P2×7R antagonist, AZ 10606120 (Figure 1A).

In order to assess if ATP cell death induction was due to apoptosis, we treated AML cells isolated from 23 AML samples with increasing doses up to 5 mM ATP for 48 h in presence or absence of P2×7R antagonist. As shown in Figure 1B, P2X7R activation by 5 mM ATP significantly increased apoptotic AML cells as compared to control (47.5 ± 7.9% vs 26.6 ± 5.8%, *p* < 0.05). To further confirm P2×7R involvement, we treated AML cells that had previously undergone to P2×7R silencing by short interfering RNAs (siRNA) (Figure 1C). Accordingly, whereas mock-nucleofected cells maintained the capability to respond to ATP stimulation (fold increase of apoptotic cells 2.3 ± 0.5, *p* < 0.05), cells transduced with anti-P2×7R siRNA failed to respond (Figure 1D), indicating that P2×7R activation is essential for apoptosis.

**Figure 2:**
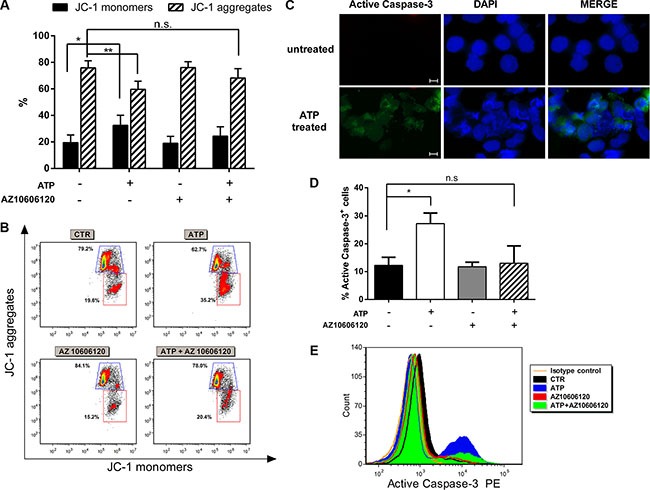
P2×7 activation induces mitochondrial stress and activation of caspase cascade AML cells were treated with 5 mM ATP with or w/o 10 μM AZ 10606120 for 48 h. (**A**) Effect of ATP on transmembrane potential in mitochondria was detected by FACS analysis. The bar graphs show the percentage of JC-1 aggregates (cells emitting red fluorescence in the FL-2 channel) and JC-1 monomers (cells emitting green JC-1 detected in the FL-1 channel) from 6 independent experiments. Data are represented as mean +/− SEM (**B**) Representative dot plots showing JC-1 staining. (**C**) Immunofluorescence analysis of activated caspase-3 (green), nuclei was counterstained with DAPI (blue). 40× magnification, scale bar 20 μm. (**D**) The histogram summarizes the percentage of activated caspase-3 from 6 independent experiments at FACS analysis. Data are represented as mean +/− SEM (**E**) Representative overlay of an independent experiment. *p < 0.05, **p < 0.01, n.s., not significant.

To better characterize apoptotic process after ATP treatment, we analyzed two specific markers of apoptosis: caspase activity and mitochondrial membrane potential (ΔΨm). To confirm mitochondrial membrane damage after 48 h ATP treatment, we stained AML cells with the cationic lipophilic dye JC-1 which accumulates as aggregates or monomers in healthy or damaged mitochondria, respectively. ATP exposure resulted in ΔΨm reduction in treated as compared to untreated AML cells as demonstrated by the increase of JC1 monomer percentage (32.6 ± 7.5% and 19.5 ± 5.8% respectively, *p* < 0.05) matched with significant decrease of JC-1 aggregates (75.9 ± 5.3% in treated cells and 59.7 ± 6.1% in untreated cells,*p* < 0.01). Such process was inhibited by the addition of AZ 10606120 (Figure 2A–2B).

Then we evaluated caspase cascade activation by analyzing the expression of caspase-3 active form. Immunofluorescence analysis revealed an increased expression of active caspase-3 in AML cells after ATP exposure (Figure 2C). Caspase-3 activation was also confirmed by flow cytometry analysis (Figure 2D–2E). The percentage of active caspase-3^+^ cells was 12.3 ± 2.0% in untreated cells and 27.3 ± 2.7% in ATP treated cells (*p* < 0.05), blockage of P2×7R by AZ10606120 restored basal level of caspase-3 activation (13.0 ± 4.4%).

Taken together our results indicate that primary AML cells undergo apoptosis after P2×7R stimulation by high ATP dose through caspase and mitochondria pathways activation.

### Bone marrow stroma does not affect ATP treatment efficacy

**Figure 3:**
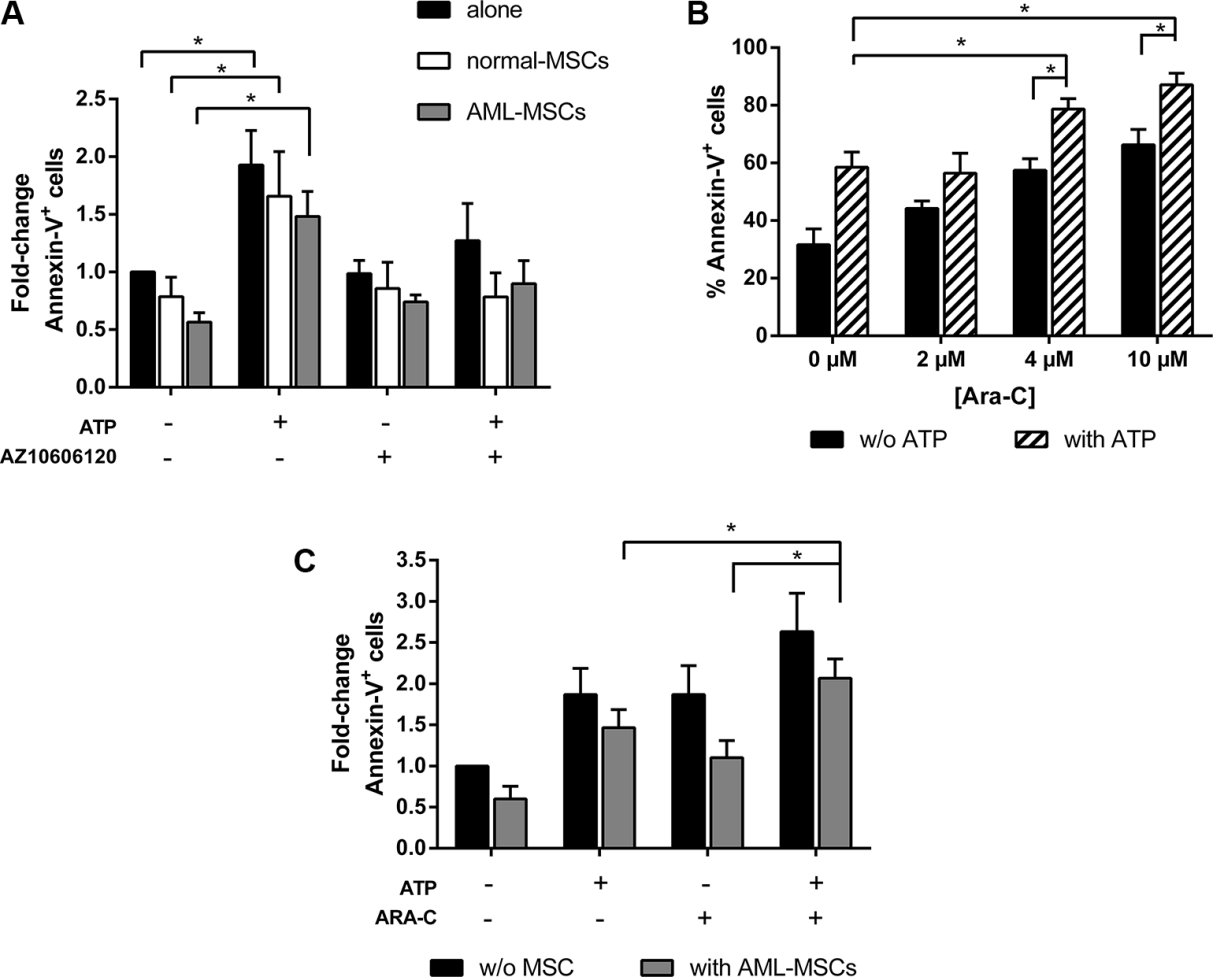
Stroma cells do not affect antineoplastic activity of ATP (**A**) AML cells were cultured alone or in presence of normal-MSCs and AML-MSCs stroma, at the ratio 10:1. After overnight, 5 mM ATP with or w/o 10 μM AZ 10606120 was added to the culture. Apoptosis induction was detected after 48 h by FACS analysis of Annexin-V^+^ cells. Results are expressed as fold change of Annexin-V^+^ cells, untreated AML cells cultured alone (39.6 ± 5.7 %) were used as reference and set as 1 (*n* = 7). (**B**) AML cells were treated for 48 h with increasing doses of ARA-C with or without 5 mM ATP and analyzed for apoptosis by flow cytometry (*n* = 6). (**C**) AML cells were cultured alone or in presence of AML-MSCs stroma, at the ratio 10:1. After overnight, 5 mM ATP with or w/o 4 μM ARA-C was added to the culture. Apoptosis induction was detected after 48 h. Results are expressed as fold change of Annexin-V^+^ cells, untreated AML cells cultured alone (Annexin-V^+^ cells: 31.6 ± 5.5%) were used as reference and set as 1. Data are represented as mean +/− SEM in all histograms (*n* = 6). *p < 0.05.

In the last decade, emerging evidence showed that bone marrow (BM) microenvironment is a key regulator of leukemia growth and its interaction with leukemia cells is one of the cause of chemoresistance [25]. In order to mimic ATP effects on leukemia BM microenvironment, we treated AML cells in presence of normal or leukemic mesenchymal stromal cells (MSCs). Both normal and AML stroma protected AML cells from spontaneous apoptosis, as expected, but did not affect ATP treatment efficacy. Compared to spontaneous apoptosis of blast cells, P2×7R activation with 5 mM ATP induced 1.9 ± 0.3, 1.7 ± 0.4 and 1.5 ± 0.2 fold increase of apoptosis in AML cells cultured alone, with normal MSCs or AML MSCs, respectively (*p* < 0.05). Blockage of P2×7R by AZ10606120 restored basal level of apoptotic cells in each group (Figure 3A).

Since P2×7R pore formation facilitates the passage of hydrophilic chemotherapeutic agents [26], we expected to potentiate the cytotoxic effect of antineoplastic drugs and to bypass stroma induced-chemoresistance, by using ATP as adjuvant. We first examined the synergistic effect of combining the cytotoxicity of cytarabine (ARA-C) with the pro-apoptotic activity of ATP. To this end, AML cells were treated for 48 h with increasing doses of ARA-C with or without ATP. As shown in Figure 3B, AML cell apoptosis was significantly higher when ATP and ARA-C were combined as compared to their use as single compound (% of Annexin-V^+^ cells: 4 μM ARA-C+ ATP 78.7 ± 5.3% vs ATP 58.6 ± 5.3% and ARA-C 59.5 ± 5.4%, *p* < 0.05). Interestingly, the addition of ATP allows to reach a high toxicity using lower doses of ARA-C. We next investigated if the observed synergistic effect was maintained also in presence of a leukemic stroma. Compared to spontaneous apoptosis of leukemic cells, we observed 2.1 ± 0.2 fold change of apoptosis in AML cells treated with ATP plus ARA-C in presence of AML-MSCs (Figure 3C). The increase was significantly higher than AML cells treated with ATP or ARA-C alone in presence of stroma (1.5 ± 0.2 and 1.1 ± 0.2, respectively, *p* < 0.05).

Taken together, our results demonstrate that ATP antineoplastic activity is maintained also in presence of mesenchymal stromal cell. Moreover, the usage of ATP as adjuvant could bypasses stroma induced-chemoresistance and potentiates ARA-C cytotoxicity.

### ATP has direct toxicity on LSCs

**Figure 4:**
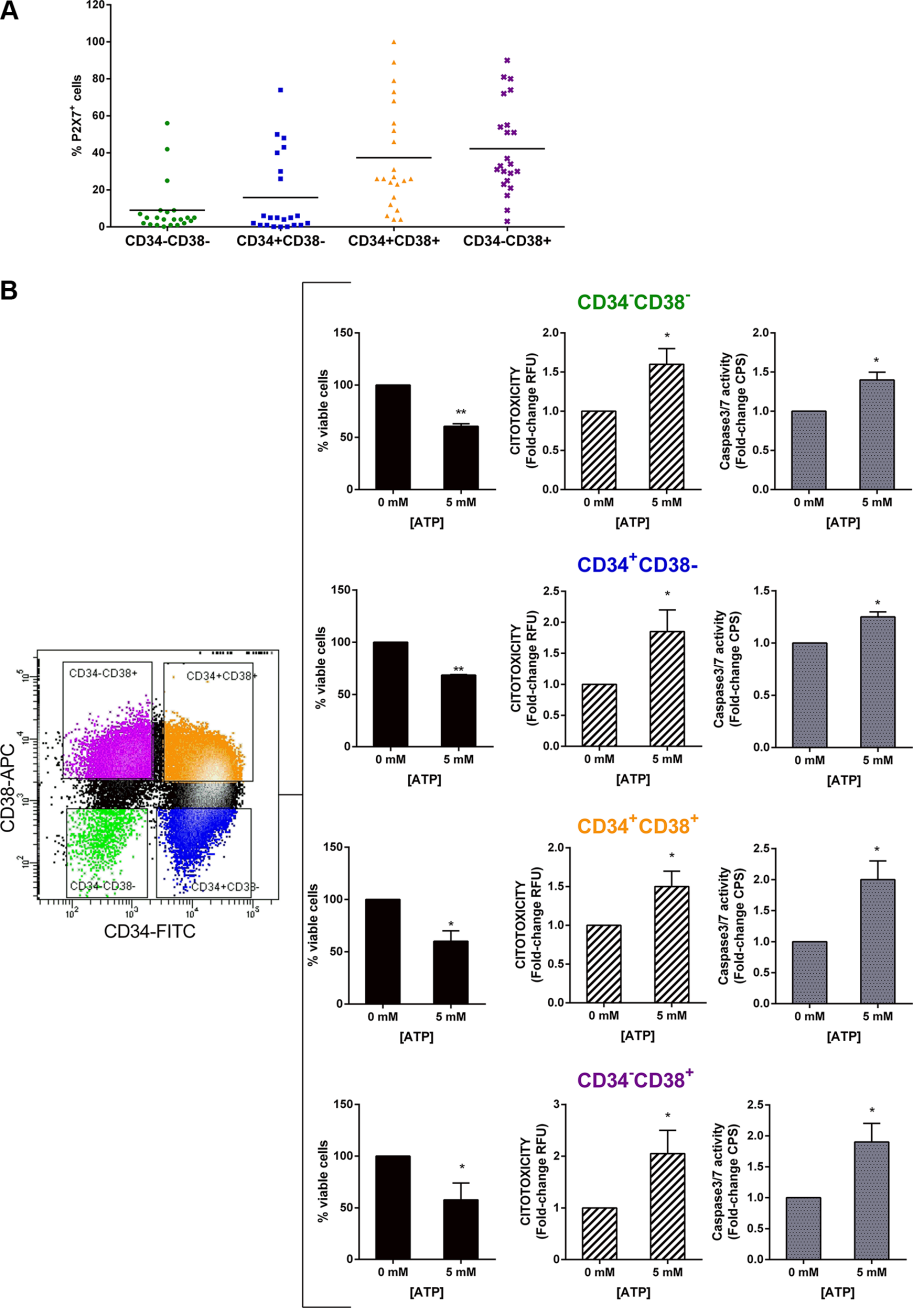
ATP exerts a direct toxicity on LSCs (**A**) Flow cytometric analysis of P2×7 expression on leukemic stem progenitor subsets (*n* = 22). (**B**) ApoTox-Glo Triplex assay showing the viability, the cytotoxicity (RFU) and apoptosis (CPS) of highly purified LSC subsets treated with 5 mM ATP for 48 h. Results from cytotoxicity and Caspase-3/7 activity assay are expressed as fold-change of RFU and CPS, respectively. Untreated cells are used as reference and set as 1. Data are represented as mean +/− SEM (*n* = 4). *p < 0.05, **p < 0.01.

Several studies [27, 28] indicate a central role of LSCs in both the genesis and the perpetuation of AML but standard chemotherapy approaches may not effectively target the LSC population. To date there are numerous studies on P2×7R expression by tumor cells, including leukemia cells, but there are no data about its expression on LSCs. In order to investigate the efficacy of ATP treatment in leukemia stem progenitor compartment, we first examined P2×7R expression in four different LSC subsets: CD34^-^CD38^-^, CD34^+^CD38^-^, CD34^+^CD38^+^, CD34^-^CD38^+^. Flow cytometric analysis of 22 AML BM samples showed that P2×7R is expressed by all populations analyzed although higher expression was seen in more differentiated progenitor cells (Figure 4A). Next, we tested ATP toxicity on LSC compartment. To this end, we purified LSC populations from 4 AML BM samples, two samples showed > 40% and the other two samples < 20% of P2×7R^+^ cells in all subsets. Then, we treated them with 5 mM ATP for 48 h. As shown in Figure 4B, ATP exposure induced a reduction of viability combined with a cytotoxicity index increase in all subpopulations analyzed. Specifically, percentage of viability and fold change of cytotoxicity index of treated cells were: 60.5 ± 2.5% and 1.6 ± 0.2 for CD34^-^CD38^-^ cells, 68.5 ± 0.5% and 1.6 ± 0.7 for CD34^+^CD38^-^ cells, 60.0 ± 10% and 1.5 ± 0.2 for CD34^+^CD38^+^ cells, 61.5 ± 27.5% and 2.1 ± 0.5 for CD34^−^CD38^+^. Similarly to total AML cells (Figure 1), increased cytotoxicity was due to induction of apoptosis as demonstrated by increased caspase-3/7 activity. Interestingly, also primitive progenitors, which express lower level of P2×7R, showed sensitivity to ATP-mediated cytotoxicity, thus suggesting that ATP may act also on cells with reduced P2×7R expression.

Taken together our results demonstrate that ATP treatment is effective at inducing toxicity not only in leukemic blasts but, more importantly, also in LSCs.

### High dose ATP does not affect normal hematopoietic stem cell compartment

**Figure 5:**
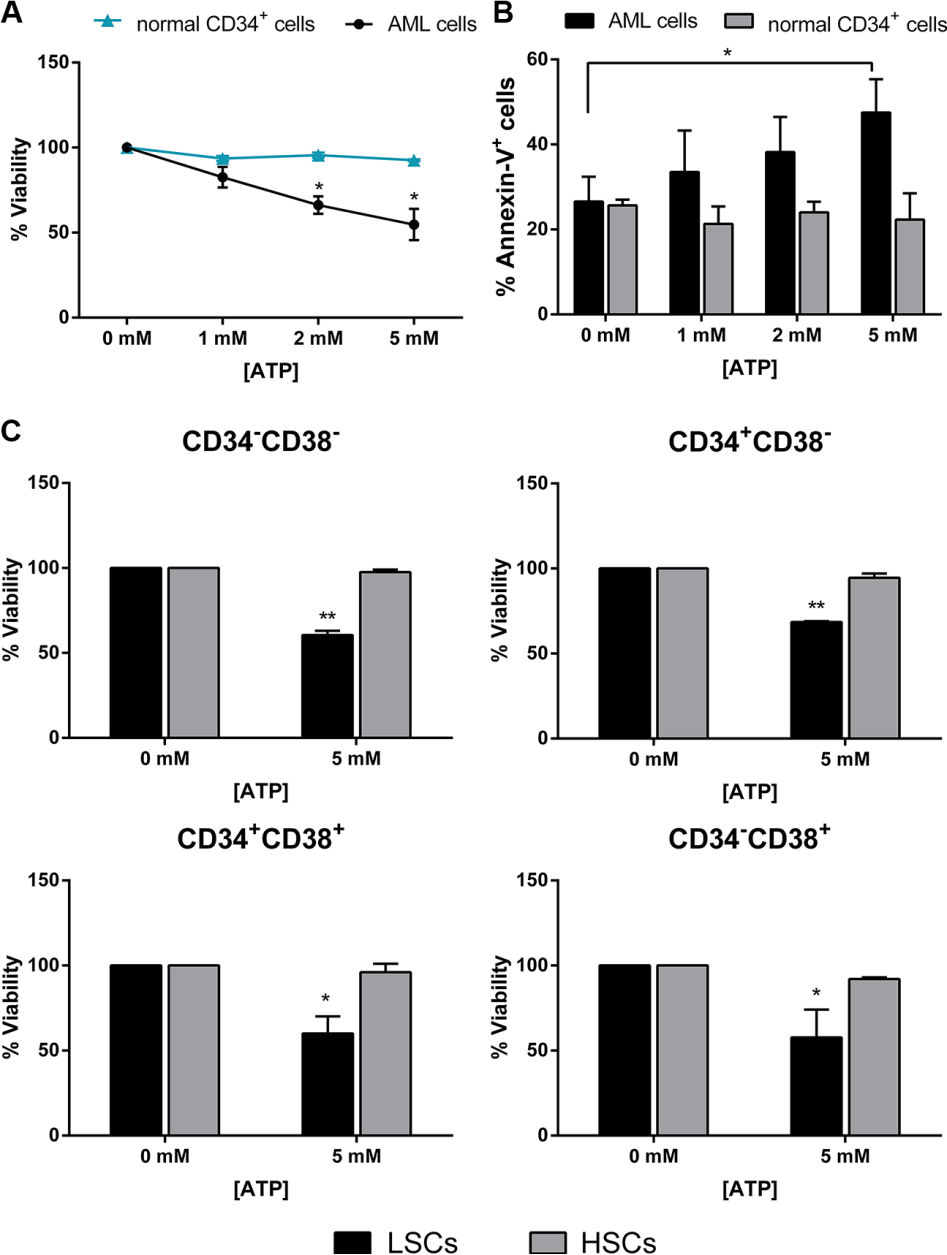
HSCs were almost entirely unaffected by ATP treatment Leukemic cells isolated from AML patients and normal CD34^+^ isolated from healthy donors were treated for 48 h with increasing doses of ATP. (**A**) CellTiter 96 Aqueous One Solution assay was used to detect viability (AML *n* = 14, donor *n* = 4) and (**B**) Annexin V/PI staining was used to detect apoptosis (AML *n* = 23, donor *n* = 4). (**C**) CellTiter 96 Aqueous One Solution assay was used to detect viability of HSC and LSC subsets treated with 5 mM ATP for 48 h (AML *n* = 4, donor *n* = 3). Data are represented as mean +/− SEM in all histograms. *p < 0.05, **p < 0.01 significant with respect to untreated cells.

Along with the identification of drugs which eradicate leukemia at the level of LSCs, minimizing the toxicity of antineoplastic agents on the normal HSC compartment represents a major task. In order to test ATP cytotoxicity on HSC compartment, normal CD34^+^ cells were treated with increasing doses of ATP, under the same culture conditions used for AML cells. Interestingly, this treatment readily reduced viability and induced apoptosis of AML cells, whereas normal CD34^+^ cells were almost entirely unaffected (Figure 5A–5B). Analysis of AML sample at the highest ATP concentration showed an average viability of 54.8 ± 9.2 % after 48h ATP exposure. In contrast, normal CD34^+^ samples showed an average viability of 92.5 ± 0.5 % when analyzed by using the same condition (*p* < 0.05). We also tested the ATP treatment on normal HSC subsets. Based on the gating strategies used for AML samples and illustrated in Figure 5, we separated four progenitor subpopulations from 3 normal BM samples and tested their ability to respond to ATP treatment. Conversely to LSC subsets, normal hematopoietic progenitors showed no variation in cell viability after high ATP dosage exposure (Figure 5C).

These results provide evidence that high ATP dosage does not impair normal stem cell compartment, which is an essential aspect for a complete hematologic recovery after therapy.

### *In vivo* ATP administration reduces leukemia cell growth

**Figure 6:**
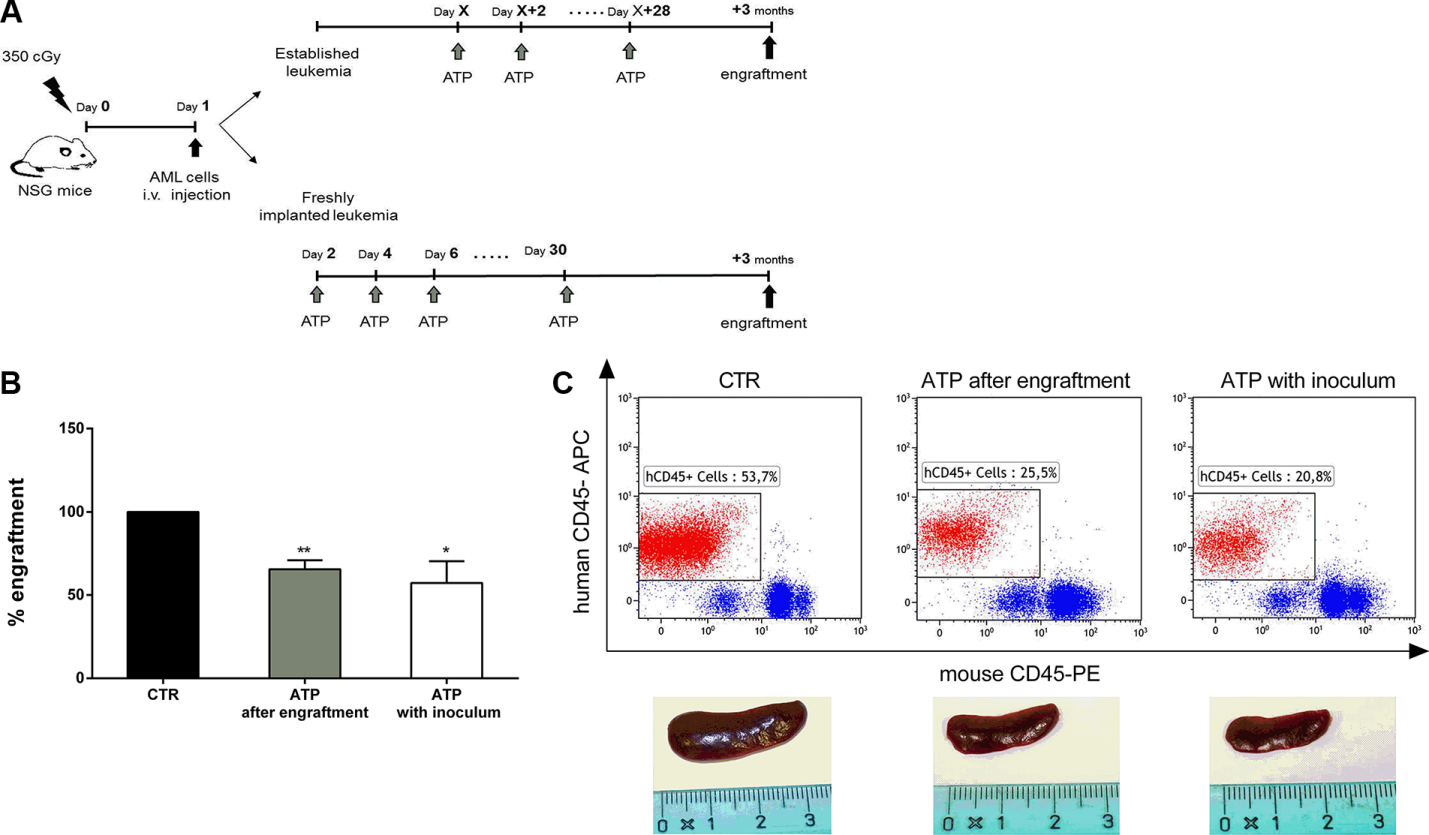
ATP administration reduces leukemia cell growth in NSG mice (**A**) Schematic representation of treatment schedule: NSG mice were injected with 1 × 10^6^ i.v. cells. In the first group, the day after human leukemia transplanted, 50 mM ATP was administrated every other day for 30 days; in the second group, the same therapeutic regimen started once AML was established. (**B**) The histogram summarizes results from four independent experiments which were performed using leukemia cells from 4 different AML patients. Results represent the percentage of human cell engraftment in mice, 3 months after the end of ATP treatment. Percentage of human engraftment in CTR mice (24.9 ± 16.9%) was used as reference and set as 100%. Data are represented as mean +/− SEM. (**C**) In upper panel, representative plots of the percentage of human tumor cells in peripheral blood samples of mice. In lower panel, spleen size of CTR vs ATP treated mice. *p < 0.05, **p < 0.01.

To asses if ATP antitumor effect observed *in vitro* also occurs *in vivo*, we investigated the pharmacologic effect of ATP administration in xenotransplanted mice. We used two experimental groups (Figure 6A). In the first one, we assessed the effect of ATP treatment on freshly implanted leukemia by administrating ATP starting the day after tumor inoculation. In the second one, the change in rate of growth of an established leukemia after ATP treatment was assessed. The same therapeutic regimen was used, but treatment was commenced once human engraftment was greater than 0.1% in peniferal blood of mice. As shown in Figure 6B, we observed a reduction of about 40% of human leukemia engraftment three months after the end of treatment, in both experimental groups (percentage of human engraftment: 57.3 ± 13.0% and 65.5 ± 5.4%, in first and second group respectively. *p* < 0.01), in agreement with the results from *in vitro* experiments. At necroscopy, control mice had enlarged spleen in comparison to ATP treated groups (Figure 6C). Treatment was well tolerated as suggested by the maintenance of body weight and the lack of toxicity primary signs, such as lethargy, ruffled fur, respiratory distress and hunchback posture (data not shown).

These results indicate that *in vivo* ATP administration is well tolerated and efficiently reduce leukemia cell growth, therefore they provide solid evidence for the further evaluation of ATP in clinical trials.

## DISCUSSION

LSCs resistance to current therapies as well as treatment-related toxicity are the main cause of failure in AML patients especially if elderly. More than 70% of AML occur in adults aged > 60 years, who are often unable to receive chemotherapy and whose prognosis is, consequently, poor [29, 30]. Therefore, the development of therapeutic approaches that target LSC population minimizing toxicity on HSCs, essential for a complete hematological recovery after treatment, is highly warranted.

There is growing interest in the therapeutic potential of purinergic signaling for the treatment of cancer [31]. After the first reports by Rapaport in 1983 who demonstrated that exogenous ATP inhibited cell growth of adenocarcinomatous pancreatic and colon cancer cells [32], subsequent papers have shown an anti-tumoral effect of extracellular nucleotides in various cancer cell types [31]. Interestingly, our recent studies reported that ATP exerts opposite effects on HSCs and AML cells [24]. However, the complete picture is probably more complicated and could be dependent upon the kind of malignancy analyzed. Purinergic receptors like P2×7R have been shown to promote cancer growth and progression in certain conditions [21, 33] while, in some case, to partially prevent cancer growth by immune infiltration [34, 35].

In the present study, we sought to investigate the use of ATP for P2×7R pro-apoptotic signaling activation specifically on LSCs. We demonstrated that AML cells exposed to high ATP dosage show a reduced viability and undergo apoptosis, as demonstrated by the increased mitochondrial permeability and by activation of caspase cascade. In line with previous reports on cell lines [22, 23], we confirmed that ATP cytotoxicity is mediated by P2×7R activation on primary leukemia cells.

BM microenvironment has an important role in several aspects of AML, including development, progression and resistance to chemotherapy [36]. To better analyze the antineoplastic effect of ATP, we tested the treatment by co-culturing AML cells with MSCs, thus mimicking BM microenvironment. Several pro-survival and anti-apoptotic signals in AML cells are activated by the stroma [37], including the phosphatidylinositol 3-kinase (PI3K)/protein kinase B (AKT)/mammalian target of rapamycin (mTOR) [38, 39] pathway (PI3K/AKT/mTOR), which weaken the response of leukemic cells to conventional chemotherapy. Since it was described that P2×7R activation by ATP leads to a concurrent blockade of the mTOR signalling [22], we hypothesized that ATP treatment could interfere in stroma-induced anti-apoptotic signals in AML cells. Interestingly, we observed that both normal and AML-derived stroma did not affect the pro-apoptotic ATP effect on leukemia blasts, suggesting that potential therapeutic treatment with ATP could bypass stroma-mediated AML resistance. Moreover, considering that P2×7R pore formation facilitates the passage of hydrophilic chemotherapeutic agents [26], we hypothesized that the addition of ATP would counteract stroma induced-chemoresistance and would potentiate the cytotoxic effect of chemotherapeutics. The principle of combined therapy is to maximize antineoplastic activity while minimizing toxic side effect of treatment. This is best attained by combining drugs with different mechanisms of action. We demonstrated that ATP combined with ARA-C significantly increases its effect on cell death and reduces the chemotherapeutic drug concentration needed to obtain high level of cytotoxicity on leukemia cells, also in presence of a stroma. These data may have interesting clinical implications for the management of frail elderly AML individuals, generally unfit to chemotherapy at standard dosage. Indeed, the use of ATP as adjuvant to conventional chemotherapeutics may allow to reduce their dosage and toxicity, while maintaining their therapautic efficacy.

Despite the accumulating evidence that P2×7R is overexpressed by tumor cells, no data are available about its expression on LSCs. We report for the first time that leukemic stem progenitor subsets express P2×7R and interestingly its activation by high ATP dose effectively affects LSCs viability, inducing apoptosis. In contrast, HSCs appear to be resistant to the same treatment. These findings clearly demonstrate that activation of the same receptor by ATP on normal and leukemic cells could selectively target LSCs and it represents a good premise for the development of new therapeutic strategies. Although the underlying mechanism of this opposite effect is not yet clarified, our hypothesis is that a differential expression of P2×7R isoform on normal and leukemic cells may occur. Nine different human splice variants of P2×7R receptor have been identified (P2×7RA-J), four of these lack the extended C-terminal tail typical of the full-length receptor (P2×7RA) [40, 41]. Among the truncated splice variants, P2×7RB exerts a trophic activity in HEK293 cells, losing the pro apoptotic function of P2×7RA. Interestingly, P2×7RB acts as dominant positive subunit by enhancing all its known functions, including apoptotic pore opening and cell growth, when co-expressed with P2×7RA [42]. Moreover, P2×7RA and P2×7RB isoforms have differential effects on cell growth and on mineralization of osteosarcoma cells [43]. We demonstrated that in normal HSCs P2×7RB is the predominant isoform (manuscript in preparation) and even if high ATP concentrations will occur, they fail to cause pore formation. The preferential expression of P2×7RB in normal CD34^+^ could be one of the mechanism by which HSCs sense the high ATP concentration in site of inflammation as growth stimulus, protecting themselves from apoptosis.

In keeping with *in vitro* experiments, we demonstrated that ATP antineoplastic effect also occurs *in vivo*. ATP administration significantly reduced human leukemia growth *in NSG mice*, without causing any adverse side effects in treated animals. The present results are the first evidence of the efficacy of ATP treatment in haematological malignancies *in vivo* and they are in line with those described in murine models of solid tumors, such as colon and pancreatic cancer [44], melanoma [45], bladder [46] and prostate [47] cancer. In our *in vivo* experiments, high ATP concentration has been used to level that exceeds the dose permitted in humans. However, since murine blood has higher ecto-ATPase activity *in vitro* compared to human blood [46] thus leading to a greater breakdown of ATP, we would expect to obtain the same therapeutic effect in patients by using a lower concentration.

Several clinical application of extracellular ATP have been reported in several fields, including oncology [48–52]. Studies from Agteresch [49, 50, 52] demonstrated the safety of ATP infusion in patient with lung cancer. ATP treatment was well tolerated and if side effects occur, they were mild and transient. In randomised trials, overall survival time of patients with advanced stage IIIB non-small cell lung cancer increased from 9.3 months in the ATP-treated vs 3.5 months for the control group. Furthermore, Beijer and colleagues showed that, in contrast to many conventional antitumor regimens, ATP infusions may have a beneficial effect on survival without negatively affecting quality of life of cancer patients affected by various solid tumors [48, 51]. It has also been demonstrated that ATP administration reduced disease activity and inflammation in a patient with active rheumatoid arthritis [53].

In conclusion, P2×7R stimulation by ATP induced a therapeutic response in blasts from AML patients and more importantly in LSCs, while did not affect HSC compartment. Moreover ATP potentiated the cytotoxic effect of chemotherapic drugs, allowing to reduce their dose. Murine model confirmed the antileukemic activity *in vivo*.

These results provide evidence that ATP would be effective and promising for developing innovative therapy for AML.

## MATERIALS AND METHODS

### Cell isolation and culture

Primary leukemic cells were obtained from peripheral or bone marrow blood of 34 AML patients at diagnosis, before treatment (Supplementary Data, Supplementary Table S1). The percentage of leukemic blasts was always > 90%. Normal mononuclear cells (MNCs) were obtained from BM healthy donors. Mononuclear cells were isolated by Ficoll-Hypaque centrifugation (Amersham Bioscience, Piscataway, NJ, USA). In some experiments, normal PB MNCs were processed by MiniMacs high-gradient magnetic separation column (Miltenyi Biotec, Bergisch Gladbach, Germany) to obtain highly purified CD34^+^ cells.

For *in vitro* studies, cells were cultured in RPMI 1640 medium supplemented with 10% FBS (GIBCO, Thermo Fisher Scientific, MA, USA) in presence of increasing dose of ATP (Sigma Aldrich, Germany) and ARA-C (Sigma Aldrich). In some experiments, cells were pre-treated for 30 min at 37°C with 10 μM AZ 10606120, a P2×7R specific antagonist (Tocris, Bristol, United Kingdom).

The pH of ATP solution was adjusted to 6.8 using NaOH for both *in vitro* and *in vivo* experiments.

The research was approved by the Ethics Committee of Policlinico S.Orsola-Malpighi, University Hospital of Bologna and each individual gave written informed consent (Ethical Committee approval code: 147/2013/O/Tess).

### Viability assay

CellTiter 96 AQueous One Solution Cell Proliferation Assay (Promega Corporation, Madison, WI, USA) was used to detect cell viability. Five × 10^5^ cells/100 μl culture medium were seeded into 96-well microplate and treated as indicated. After culture, CellTiter 96 AQueous One Solution reagent was added to each well and the microplate was incubated for 4 h at 37°C in 5% humidified CO_2_. Optical density value was measured by an ELISA plate reader (Multiskan Ex, Thermo Electron Corporation, San Josè, CA, USA) at a wavelength of 492 nm. Each variant group was performed in triplicate wells.

### Apoptosis

Apoptosis was measured with Annexin-V-FLUOS Staining Kit (Roche, Mannheim, Germany) followed by flow cytometric analysis (BD AccuriTM C6, BD Becton Dickinson, NJ USA. FCS Express 4 Software, De Novo Software, Los Angeles, CA USA). After the indicated treatment, cells were harvested and stained according to manufacturer's instructions. Briefly, 1 × 10^6^ cells were resuspended in 100 μl Incubation Buffer, stained with 2 μl FITC-conjugated Annexin-V and 2 μl propidium iodide (PI) for 10 min at room temperature and analyzed. Results were compared to the apoptotic rate of untreated cells.

### siRNA transfection of AML cells

AML cells were transfected with 350 nM SMARTpool ON-TARGETplus P2RX7 siRNA (Dharmacon, Lafayette, CO), using standard Nucleofector Amaxa Thecnology (Amaxa Biosystem, Germany) [54]. Briefly, 2 × 10^6^ AML cells were transfected by using the U08 program. The target sequences were: i) GCGGUUGUGUCCCGAGUAU, ii) GGAUCCAGAGCAUGAAUUA, iii) GCUUUGCUCUG GUGAGUGA, iiii) GGAUAGCAGAGGUGAAAGA. ON-TARGETplus Non-targeting Control Pool designed and tested for minimal targeting of human genes was used as negative control (Dharmacon). After 24 h incubation, AML cells were treated with 5 mM ATP with or without 10 μM AZ 10606120 for 24 h.

### Active caspase-3 expression

Flow cytometric analysis of active caspase-3 was performed using Fixation Permeabilization kit (BD) for intracellular staining and human active Caspase-3-PE monoclonal antibody (BD), according to protocol's instruction. BD AccuriTM C6 cytometer and FCS Express 4 Software were used.

For immunofluorescence staining, cells were fixed in 4% parafolmaldehyde and permeabilized with 0.25% Triton. The staining was performed using anti-Human CASP3 monoclonal antibody (Invitrogen, CA USA) and anti-Rabbit FITC secondary antibody (Dako, Denmark). Cell were examined under fluorescence Axiovert microscope with a CCD camera (Zeiss, Germany)

### Mitochondrial membrane potential measurement

The variation in ΔΨm was investigated using the BD^™^ MitoScreen Kit (BD) according to the manufacturer's instruction. Briefly, 1 × 10^6^ cells were harvested from the suspension cultures, washed twice with PBS and incubated with JC-1 solution for 15 min at 37°C. JC-1 monomers or aggregates were analyzed by flow cytometry (BD AccuriTM C6 cytometer and FCS Express 4 Software).

### Co-culture of AML cells with MSCs

Normal or AML MSCs, isolated as previously described [55], were seeded in 96 well plates 1 × 10^4^/well, AML cells were added to the confluent layer at 10:1 ratio. After overnight incubation, cells were treated as indicated. After 48 h treatment, AML cells were harvested for analysis, leaving MSC stroma intact.

### Flow cytometry and cell sorting

P2×7R expression in LSC subsets was analyzed by flow cytometry (BD AccuriTM C6 cytometer and FCS Express 4 Software). BM cells from 22 AML samples were labelled with an anti-P2×7R FITC (Alomone Labs, Israel), anti-CD34 APC (BD) and Anti-CD38 PE (BD) monoclonal antibodies.

Leukemic and normal stem/progenitor cell subset were purified by cell sorting strategy. BM MNCs were stained with human CD34-FITC and CD38-APC monoclonal antibodies (BD) and sorted on FACSAria (BD). Aliquots of sorted populations were reanalyzed to assess their purities, which were always found to be greater than 98%.

### Caspase-3/7 activity assay

ApoTox-Glo^™^ Triplex Assay (Promega) was used to assess cytotoxicity and caspase-3/7 activity in LSC subsets. Cells were plated in 96-well plate (2,000 cells/100 μl /well) and treated with 5 mM ATP for 48 h. At the end of culture, according to protocol's instruction, we added 20 μl of Viability/Cytotoxicity Reagent to each well. After 30 min incubation at 37°C, cell viability and cytotoxicity (wavelength 400_Ex_/505_Em_, 485_Ex_/520_Em_ respectively) were assessed by measuring fluorescence with Victor2 Microplate Reader (Perkin Elmer, MA USA). After the measurement of cell viability, the caspase-Glo 3/7 reagent was added into each well, the plates were briefly mixed by an orbital shaker and incubated for 30 min at 37°C. Caspase-3 activation was determined by measuring luminescence with Victor2 Microplate Reader. Each variant group was performed in triplicate wells.

### Xenotransplant experiments

Experiments were carried out on nonobese diabetic severe combined immunodeficient (NOD/SCID) interleukin-2 receptor g (IL-2Rg)–null (NSG) mice, 6 to 8-weeks-old. NSG mice were bred and housed under pathogen-free conditions in the animal facilities at the European Institute of Oncology–Italian Foundation for Cancer Research (FIRC) Institute of Molecular Oncology (IEO-IFOM, Milan, Italy). All animal experiments were done in accordance with the Italian Laws (D.L.vo 26/2014 and following additions), and institutional guidelines, and were approved by the Italian Ministry of Health. For induction of human leukemia, 1 × 10^6^ AML cells were injected intravenously through the lateral tail vein of sublethally irradiated mice. Primary cells from 4 different AML patients were used.

Two experimental treatment design were used. In the first group, one day after cell transfer 50 mM ATP in 0.25 ml sterile saline was administered for 30 days, every other day by i.p. injection. In the second group, the same therapeutic regime was used as before, but the treatment started once AML was established. A control group (CTR) received i.p. an equivalent volume of the vehicle (sterile saline solution). Five mice were randomly picked and assigned to each experimental group. Human cell engraftment in the peripheral blood from tail vein was investigated by flow cytometry. Cells were stained with anti-human CD45-APC (Beckman-Coulter, CA, USA) and anti-mouse CD45-PE (BD) antibodies. After red cell lysis, cell suspensions were evaluated by a FACSCalibur (BD). Seven-aminoactinomycin D (7AAD; Sigma-Aldrich) was used to enumerate viable, apoptotic and dead cells.

### Statistical analysis

Results are expressed as means ± SEM

Depending on experimental conditions, statistical analysis will be performed using *t* test or ANOVA followed by a *post hoc* test (Bonferroni or Dunnet’s) (GraphPad Prism, version 6.03). *P* < 0.05 was considered statistically significant.

## SUPPLEMENTARY MATERIALS AND TABLES


