# Supplementary material for: Extracellular ATP induces apoptosis through P2X7R activation in acute myeloid leukemia cells but not in normal hematopoietic stem cells
Source: Oncotarget. 2016 Dec 13;8(4):5895–908. doi: 10.18632/oncotarget.13927 (PMC5351599; doi:10.18632/oncotarget.13927)
Supplement: Supplementary file 1 [file oncotarget-08-5895-s001.pdf]

Extracellular ATP induces apoptosis through P2X7R activation in acute myeloid leukemia cells but not in normal hematopoietic stem cells

Supplementary Materials

Supplementary Table S1: Patient characteristics

# PT	Cell source	FAB	Molecular abnormalities	Karyotype	WBC, cells/µL	Sex	Age
1	BM	M4	NPM^-, FLT3-	t(16;16)	95,450	F	46
2	PB	M2	NPM^+, FLT3-ITD^+	NA	64,250	M	70
3	BM	M4	NPM^+, FLT3+	t(4)	129,000	F	60
4	BM	NA	NA	Normal	15,800	F	59
5	PB/BM	M1	NPM^-, $FLT3^+$, $DNMT3A^-$	Normal	57,400	F	64
6	PB	M2-M4	NA	inv(16)	14,000	M	68
7	BM	NA	NPM^+, $FLT3^-$	NA	NA	M	75
8	BM	M4-M5	NPM^+, $FLT3^+$	Normal	50,000	M	63
9	BM	M3	NPM^-, $FLT3^-$, PML/RARα	t(15;17)(q22;q12)	1,100	M	39
10	PB/BM	M4	NPM^+, $FLT3^-$	Normal	25,300	F	63
11	PB	M1-M2	NPM^+, $FLT3^-$	Normal	21,300	F	66
12	BM	M0-M1	NPM^-, $FLT3^-$, DNMT3A-	Complex	8,000	M	21
13	PB	M2-M4	NPM^+, FLT3-ITD^+; FLT3-TKD^- DNMT3A+	Normal	46,700	F	68
14	BM	M3	NPM^-, $FLT3^-$, PML/RARα	t(15;17)(q22;q12)	8,060	M	43
15	PB	M3	NPM^+, $FLT3^-$, PML/RARα	t(15;17)	0,650	M	69
16	BM	M1	NPM^-, $FLT3^-$, $DNMT3A^-$	Complex	2,800	M	48
17	BM	M1	$FLT3^-$$ITD^+$, NPM^-	t(6;9)(p23;q34)	29,670	F	46
18	BM	M5	NPM^-, $FLT3^-$	Complex	NA	M	17
19	PB	M1	NPM^-, $FLT3^+$	Normal	35,830	M	57
20	PB	NA	NA	NA	11,800	M	90
21	PB	M0-M1	NPM^+, FLT3-ITD^-	Normal	189,500	M	64
22	PB/BM	M4	NPM^-, $FLT3^-$, $DNMT3A^-$	inv (16)	11,300	M	55
23	PB	M1	NPM^-, $FLT3^-$	t(8;21)	26,000	M	54
24	PB	M3	NPM^-, $FLT3^-$	t(15,17)	1,250	F	53
25	PB	M1	NPM^+, $FLT3^+$	Normal	95,000	F	51
26	PB/BM	M0-M1	NA	+(8)	4,280	M	68
27	PB/BM	M0	NPM^-, $FLT3^-$	t(6;11)	100,000	M	19
28	PB	M0	NPM^+, FLT3-ITD^+	Normal	108,600	F	67
29	BM	M1	NPM^+, $FLT3^-$	Normal	3,660	F	36
30	PB	M3	PML/RARα	t(15,17)	99,300	F	77
31	BM	M2	$FLT3^-$	Normal	1,580	M	73
32	PB	M1	NPM^+, FLT3-ITD^+, FLT3-TKD^+,	Normal	103,000	F	34
33	BM	M1	$FLT3^-$, NPM^-	Normal	37,200	F	38
34	PB	NA	NPM^-, FLT3-ITD^+	Normal	88,680	M	69

Legend: PB, peripheral blood; BM, bone marrow; NPM, nucleophosmin; FLT3 ITD (TDK), fms-like tyrosine kinase 3 internal tandem duplication (tyrosine kinase domain mutation); DNMT3A, DNA-methyltransferase 3A; NA, Not available.
